# Anatomical damage caused by *Bacillus thuringiensis* variety *israelensis* in yellow fever mosquito *Aedes aegypti* (L.) larvae revealed by micro-computed tomography

**DOI:** 10.1038/s41598-023-35411-1

**Published:** 2023-05-30

**Authors:** Javier Alba-Tercedor, Susana Vilchez

**Affiliations:** 1 Department of Zoology, Faculty of Sciences, University of Granada, 18071 Granada, Spain; 2 Institute of Biotechnology and Department of Biochemistry and Molecular Biology I, Faculty of Sciences, University of Granada, 18071 Granada, Spain

**Keywords:** Biological techniques, Microbiology, Zoology, Entomology

## Abstract

With micro-computed tomography techniques, using the single-distance phase-retrieval algorithm phase contrast, we reconstructed enhanced rendered images of soft tissues of *Aedes aeqypti* fourth instar larvae after *Bti* treatment. In contrast to previous publications based on conventional microscopy, either optical or electron microscopy, which were limited to partial studies, mostly in the form of histological sections, here we show for the first time the effects of *Bti* on the complete internal anatomy of an insect. Using 3D rendered images it was possible to study the effect of the bacterium in tissues and organs, not only in sections but also as a whole. We compared the anatomy of healthy larvae with the changes undergone in larvae after being exposed to *Bti* (for 30 min, 1 h and 6 h) and observed the progressive damage that *Bti* produce. Damage to the midgut epithelia was confirmed, with progressive swelling of the enterocytes, thickening epithelia, increase of the vacuolar spaces and finally cell lysis, producing openings in the midgut walls. Simultaneously, the larvae altered their motility, making it difficult for them to rise to the surface and position the respiratory siphon properly to break surface tension and breathe. Internally, osmotic shock phenomena were observed, resulting in a deformation of the cross-section shape, producing the appearance of a wide internal space between the cuticle and the internal structures and a progressive collapse of the tracheal trunks. Taken together, these results indicate the death of the larvae, not by starvation as a consequence of the destruction of the epithelia of the digestive tract as previously stated, but due to a synergic catastrophic multifactor process in addition to asphyxia due to a lack of adequate gas exchange.

## Introduction

*Bacillus thuringiensis* (*Bt*) was first discovered in 1901 by Shigetane Ishiwata who isolated a bacterium from dead silkworm larvae while he was investigating the cause of the so-called “sotto disease” (sudden-collapse disease). He named the bacterium *Bacillus sotto*^1^. Several years later, Ernst Berliner isolated a related strain from dead Mediterranean flour moth larvae found in a flour mill in Thuringia, and thereafter appropriately named the bacterium *B. thuringiensis.* This author also observed that a solution of crystallized *Bt* toxins was highly effective against certain crop pests^2–4^.

*Bt* is a Gram-positive spore-forming bacterium found all over the globe and in all tested ecosystems^5^. During sporulation, *Bt* strains synthesize crystal (Cry) and cytolytic (Cyt) protein toxins called δ-endotoxins as parasporal bodies, which are toxic to many insects^6,7^. It has been shown that when insect larvae ingest these protein crystals, they are solubilized by the alkaline environment of the midgut and protoxins are activated by the digestive enzymes causing pores in the cell membrane of the digestive tract, with lethal consequences for the insects, i.e.: Refs.^8,9^.

The first commercial production of *Bt* as an insecticide was reported in 1938 in France and sold under the name “Sporéine”, and since then its use in the development of advanced products has been continuously increasing^10^. Angus, using silkworm larvae (*Bombyx mori*) and a strain of the *Bt* subspecies *sotto*, was the first to prove that the Cry toxin was the main insecticidal agent^11^, and afterwards it was demonstrated that the gut epithelium was the site of action of the δ-endotoxins^12^. Nowadays, the Cry proteins have been tested to target diverse species of different insect orders and some other invertebrates such as mites and nematodes^13^. Although there is a long-standing controversy as to whether there are risks of long-term effects on ecosystems from the indiscriminate release of *Bt* in nature^14–16^, *Bt* products are considered a much better alternative than chemical insecticides given their specificity and biodegradability.

Since the isolation of the *Bt* variety *israelensis* (*Bti*), whose paraspores have a strong pathogenic action toward mosquito larvae^14,15^ (in addition to black fly and chironomid larvae), its use for mosquito control, and the number of publications, has increased exponentially^16^.

Although there are numerous studies on the mechanisms that allow its biocidal action, few studies have shown anatomical-histological damage^17^. Nevertheless, the midgut histopathology and pathogenesis of *Bti* toxins for several species of Culicidae mosquito larvae have been reported^18–26^, including the yellow fever mosquito^19–21,27,28^.

To assess the damage caused by *Bti*, it is important to be able to compare with the anatomy of healthy larvae. There are several studies on this topic, starting with the classical study on mosquito anatomy by Snodgrass^29^ and the extraordinary compilation by Christophers^30^, and a recent study with a histological characterization of the midgut of healthy *A. aegypti* larvae^31^.

After the isolation^15^ and characterization of a strain of *Bt* whose parasporal inclusions had strong pathogenic power for Culicidae larvae^14^, cytological studies on the histopathological effects of *Bti* in *A. aegypti* began^18,19^. The pioneering work by Charles, first with optical microscopy^19^ and then with transmission electronic microscopy^20^, was the first to demonstrate the damage caused by *Bti* at the tissue level, mainly in the digestive tract.

In contrast to previous publications, based on conventional microscopy, either optical or electron microscopy, which were limited to partial studies mostly in the form of histological sections, in this work, and thanks to micro-CT, it has been possible to show the internal anatomy of a *Bt* treated insect in its entirety for the first time. Using 3D rendered images it was possible to study not only the structures in sections but also as a whole, and by comparing the anatomy of healthy larvae and the changes undergone after being exposed to *Bti* for 30 min, 1 h and 6 h, the progressive damage that the *Bti* produces has been described.

## Materials and methods

### Bacterial strains growth conditions

The bacterial strain used in this work was *B. thuringiensis* var. *israelensis* 4Q5 (*Bti* 4Q5), from the *Bacillus* Genetic Stock Center at the Ohio State University. *Bti* 4Q5 was cultured in 50 ml of T3 medium^32^ at 30 °C and under aerobic conditions (200 rpm) for 72 h until complete sporulation was observed (10^7^ spores/ml). The culture was centrifuged at 4000 *g* for 20 min, and the pellet was washed three times and resuspended in 5 ml of Milli-Q water (22 mg spore crystal suspension/ml). The obtained spore and crystal suspension was kept at 4 °C until used.

### Larval rearing and bioassay

After arrival, the eggs were placed in a glass container with dechlorinated tap water and ground commercial dry cat food. The container was incubated in an insect room (25 °C ± 2 °C, 65% humidity and a photoperiod of 16 h:8 h light:dark). Under these conditions, eggs hatched within two days. Cat food was supplied when necessary. *Aedes aegypti* early fourth instar larvae were used for the bioassay with *Bti* 4Q5. The remaining larvae, not used in the bioassay, were killed by adding bleach to the rearing container.

Ten *A. aegypti* larvae were placed in a 30 ml plastic tube containing 10 ml of dechlorinated tap water and dry cat food and kept in the insect room under the same conditions as described previously. One hundred microliters of the previously described *Bti* 4Q5 spore and crystal suspension was added to the larvae. Two larvae were withdrawn at different times (30 min, 1 h and 6 h) with the use of a plastic Pasteur pipette and placed into plastic tubes with 5 ml of 70% ethanol. After the time course, larvae were fixed and treated for microtomography as described below. The 30 min larvae were alive, and the larvae withdrawn 1 h and 6 h after the start of the bioassay were dead.

### Specimen preparation and CT scanning conditions

**Figure 1:**
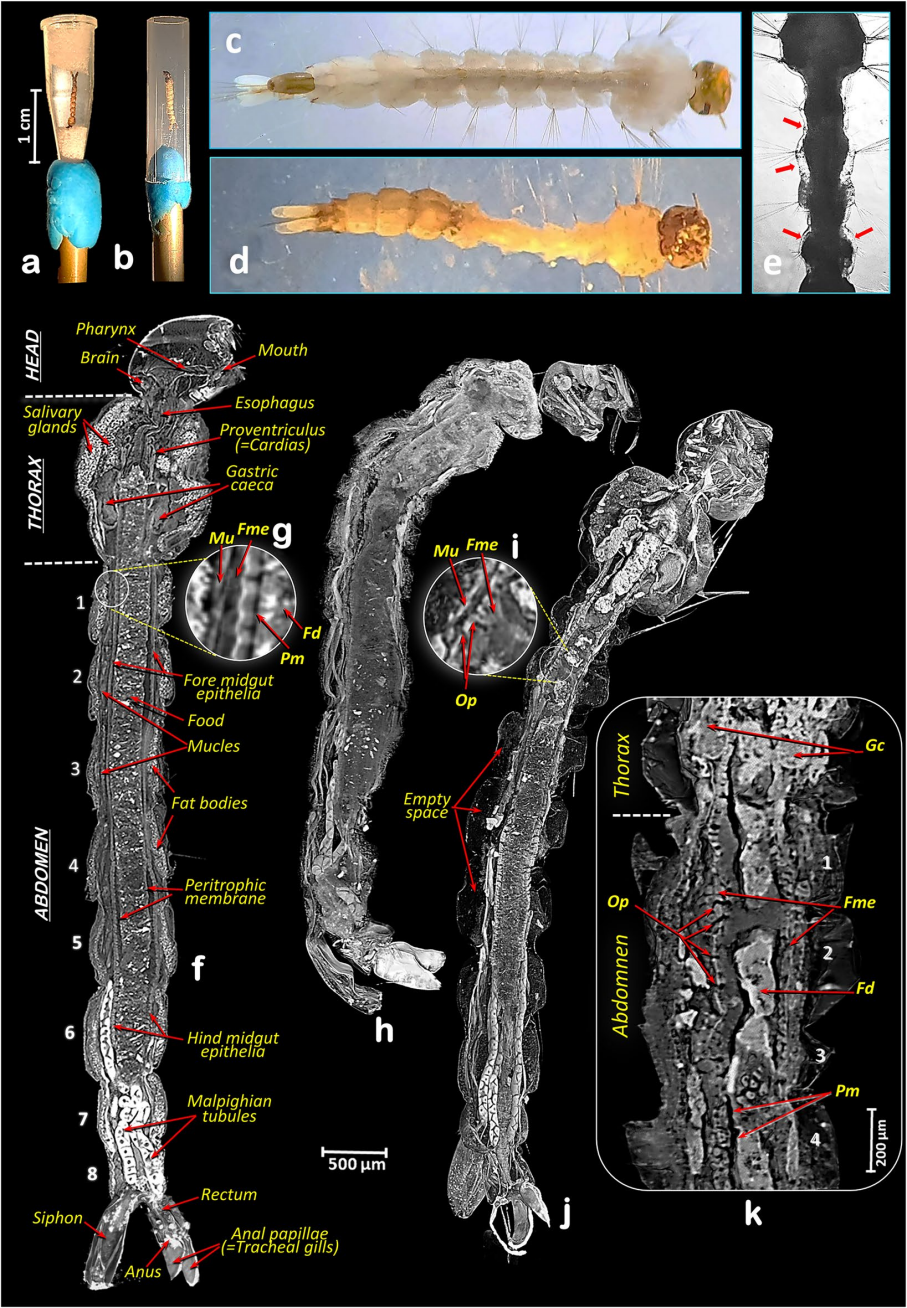
Micro-CT 3D rendered images with sagittal sections of fourth instar *A. aegypti* larvae. Mounted samples ready for scanning (**a,b**) and ethanol preserved larvae (**c–e**). Deformation (**d**) after exposition to *B. thurigiensis* var. *israelensis* (*Bti*) and empty space created, marked with red arrows (**e**). Control healthy larva (**c,f,g**). Larvae after different exposure times to *Bti *(**d,e,h–k**): 30 min (**d,e,h**), 1 h (**i,j**), 6 h (**k**). Details of the fore midgut epithelia and surrounding muscles (**g,i**). Note deformation (**d**) and empty space created (**e**), and the progressive cell lysis effect caused by *Bti*, which is observed in the form of openings in the midgut epithelia. Abdominal segments are numbered. *Fd* food, *Fme* fore midgut epithelia, *Gc* gastric caeca, *Mu* muscles, *Op* midgut epithelia openings, *Pm* peritrophic membrane.

For the microtomographic study, two larvae, already preserved in 70% ethanol, were taken from each bioassay with increasing *Bti* exposure times, and dehydrated with increasing ethanol concentrations (80%, 90%, 100%) for 30 min each at room temperature. Before scanning, the larvae were stained in a solution of 1% iodine in absolute 100% ethanol for 24 h, submerged in hexamethyldisilazane (HMDS) for 12 h and air-dried overnight. Specimens were scanned either into a 0.2 ml Eppendorf tube (which was attached to the specimen holder with plasticine, and larvae fixed inside with Basotect^®^ [melamine resin foam, created by the Chemical Company BASF], a material easy to remove digitally^33^ [Fig. 1a]) or glued with cyanoacrylate to the tip of a nylon fishing line (200 µm diameter) and covered with a plastic straw to avoid any movement induced by the air refrigerating current during the scan process (Fig. 1b). A SkyScan 1172 desktop high-resolution microtomograph, upgraded to have a Hamamatsu L702 (100/250) source and a Ximea 11Mp camera was used. The scanning parameters were set up as follows: Isotropic voxel size = 0.54 µm; Source voltage = 48 kV, Source current = 49 µA, Image rotation step = 0.53° (0.2 for the 6 h larvae), 180° rotation scan and no filter. To be able to capture the whole length of the larvae it was necessary to perform 5–7 connected oversize scans. The resulting Tiff images were reconstructed with the recent Bruker micro-CT’s NRecon software (v.2.0.0.5) using the single-distance phase-retrieval algorithm described by David Paganin et al.^34^, which permits phase contrast reconstructed enhanced images of soft tissues.

**Figure 2:**
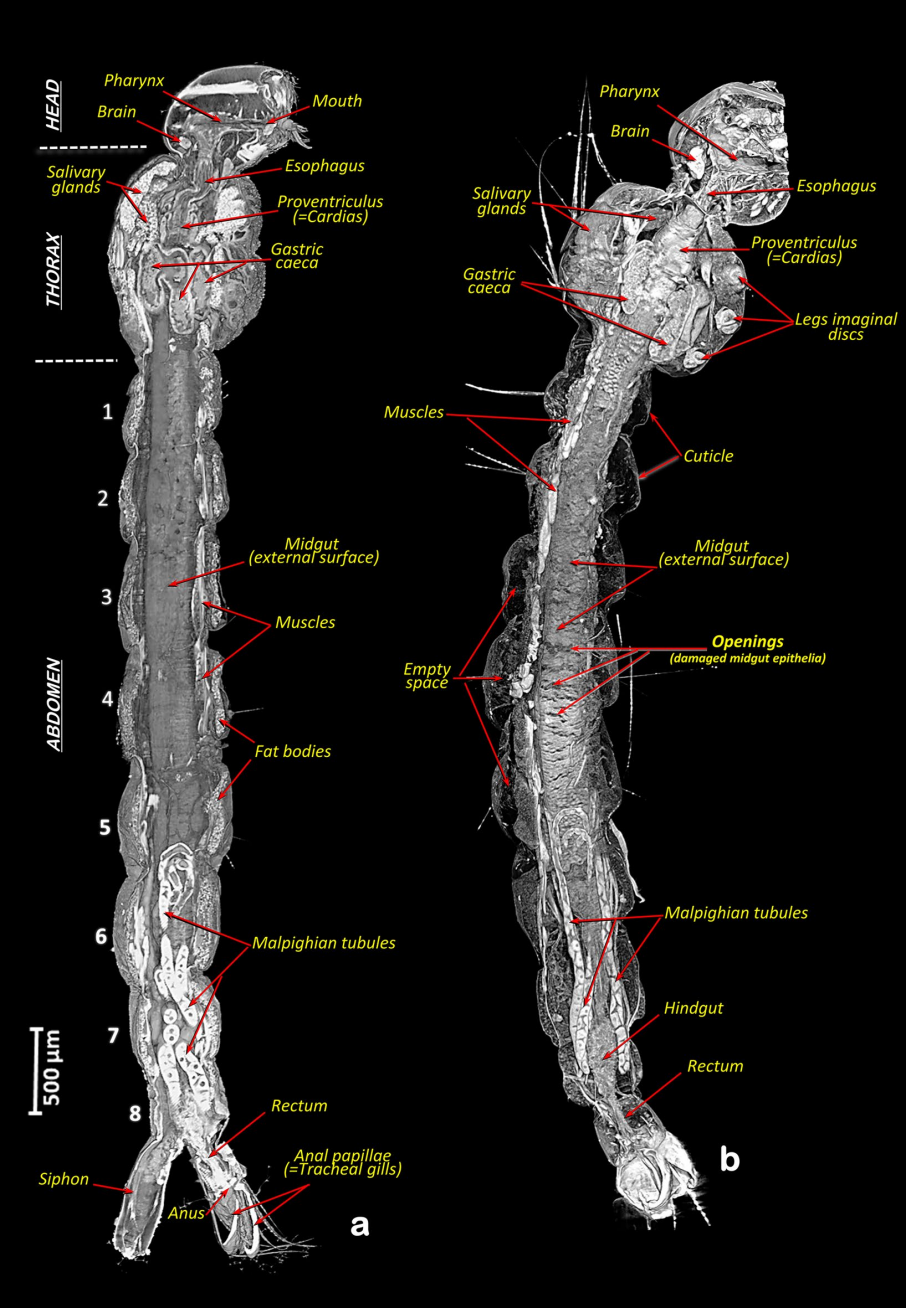
Micro-CT 3D rendered images with sagittal sections of fourth instar *A. aegypti* larvae showing the internal anatomy to visualise the external surface of the digestive tract epithelia. Control healthy larva (**a**) and larva after 1 h exposure to *Bti* (**b**). Note in b the damage caused by the *Bti*, which can be seen in the form of openings and empty space created.

**Figure 3:**
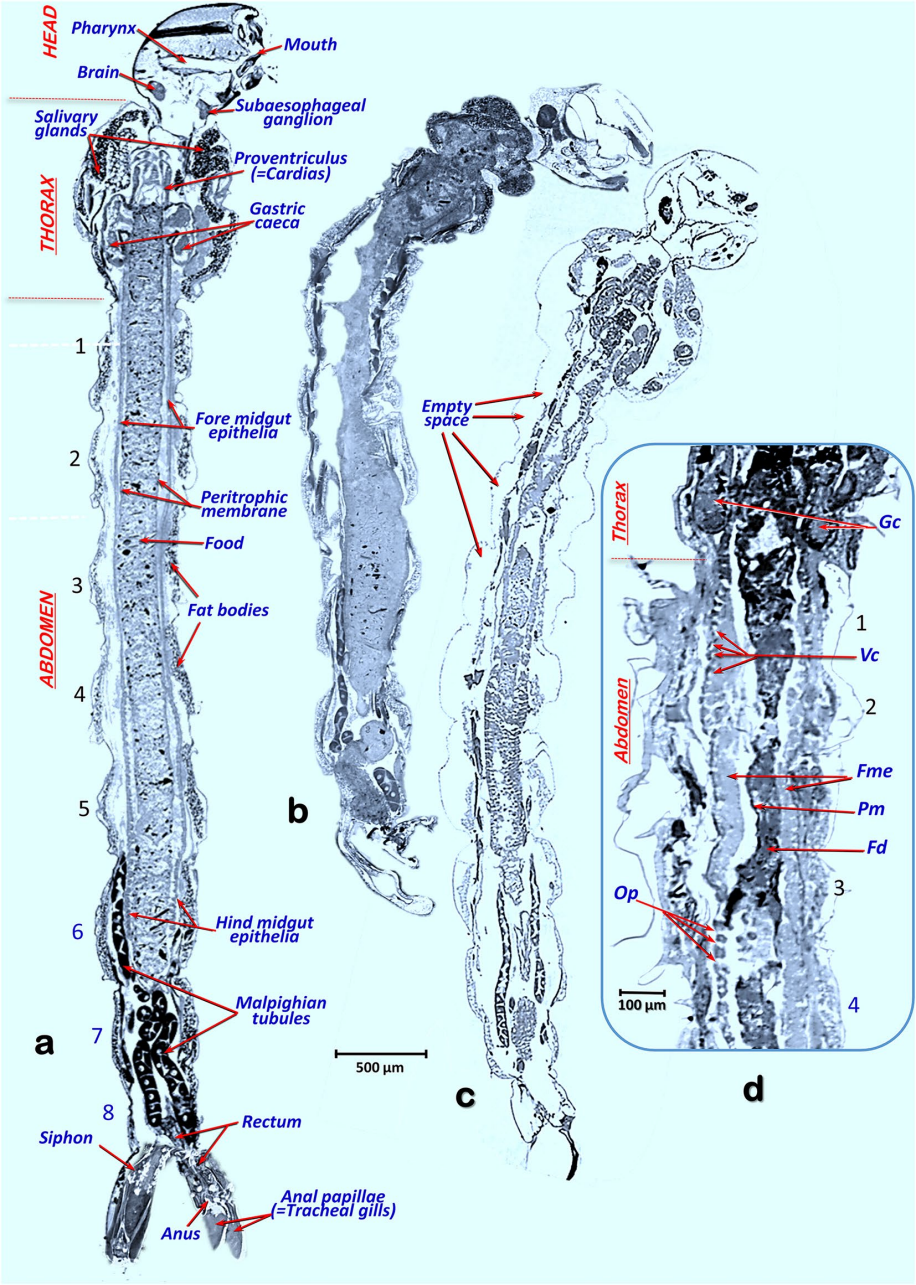
Micro-CT slice rendered images with sagittal sections of fourth instar *A. aegypti* larvae, comparable to those obtained in the literature using histological microscopy techniques, showing the internal anatomy. Control healthy larva (**a**). Larvae after different exposure times to *Bti* (**b–d**): 30 min (**b**), 1 h (**c**) and 6 h (**d**). Note empty space created and the cell lysis effect caused by *Bti*, which is observed in the form of openings in the midgut epithelia, an increase in vacuoles and a progressive swelling of the midgut cell epithelia, which is observed as an increase in thickness. *Fd* food, *Fme* fore midgut epithelia, *Gc* gastric caeca, *Mu* muscles, *Op* midgut epithelia openings, *Pm* peritrophic membrane, *Vc* vacuoles.

**Figure 4:**
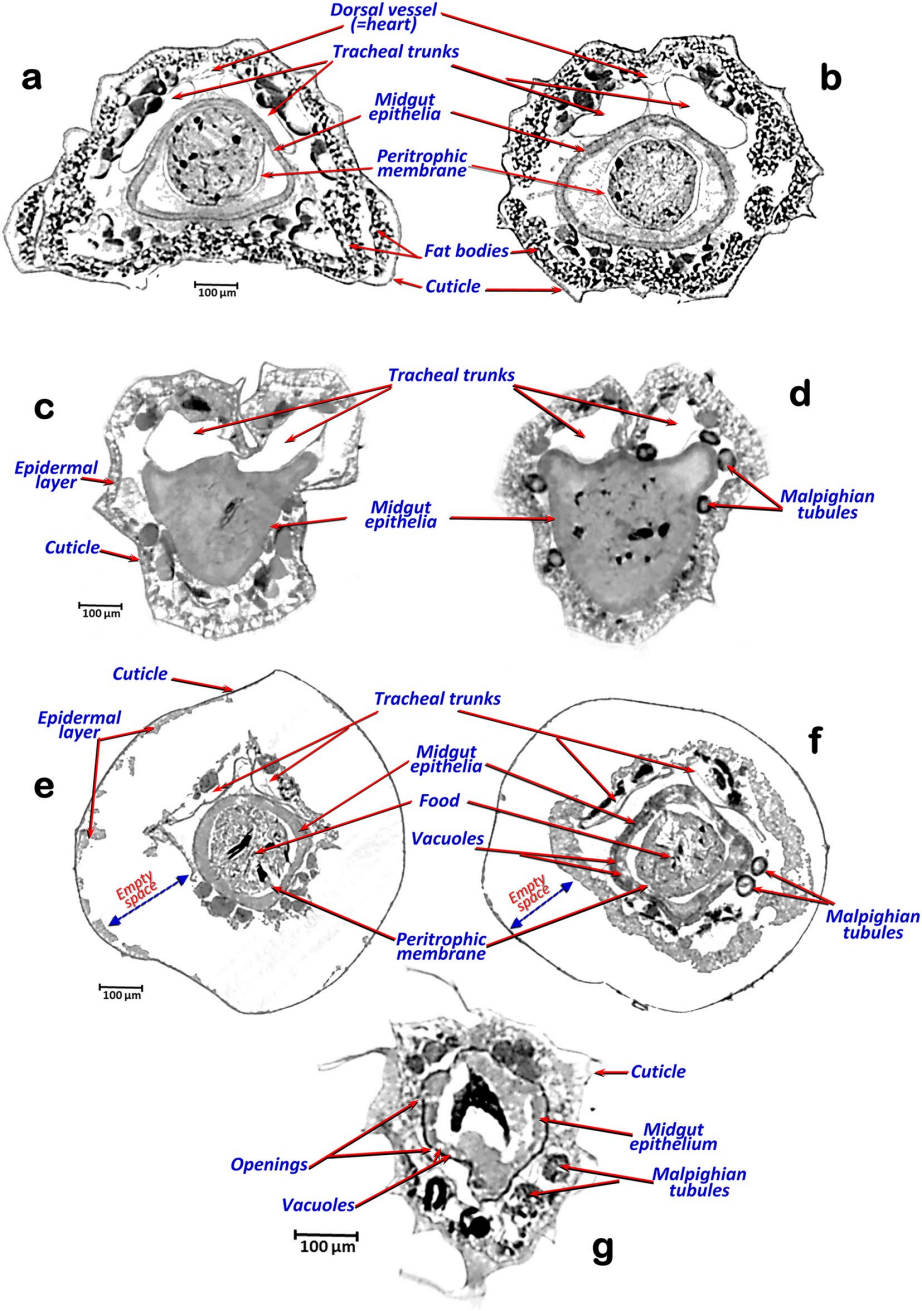
Micro-CT slice-rendered images with cross-sections of fourth instar *A. aegypti* larvae at level of the third (**a,c,e**) and fifth abdominal segments (**b,d,f,g**). Control healthy larva (**a,b**). Larvae after different exposure times to *Bti* (**c–g**): 30 min (**c,d**), 1 h (**e,f**) and 6 h (**g**). Note empty space created and the cell lysis effect caused by *Bti*, which is observed in the form of openings in the midgut epithelia, an increase in vacuoles, a progressive swelling of the midgut cell epithelia (observed as an increase in thickness) and a progressive collapse of the tracheal trunks.

The Bruker micro-CT’s Skyscan software CTAnalyser v.1.20.8.0 was used for the primary ‘cleaning’ process. The resulting images were reoriented with DataViewer v.1.6.0.0 (used to get sliced rendered images of Figs. 3 and 4), and CTvox v.3.3.1 was used to get 3D rendered images of Figs. 1f–k and 2 and the Supplementary Video S1, as previously described^35^.

## Results

The rendered micro-CT images of larvae show the main anatomical structures and organs (Figs. 1f–k and 2 and Supplementary Video S1). Thus, in addition to the external structures of the head, thorax and abdomen, the internal anatomical details are shown in detail, with the brain, the muscles, the fat bodies and the digestive tract (mouth opening, pharynx, esophagus, proventriculus, gastric caeca, midgut, rectum and the position of the anal opening). Inside the digestive tract, the surrounding peritrophic membrane and the epithelial layer can be seen. In addition, the salivary glands, the Malpighian tubules (Figs. 1f,h,j; 2, 3 and 4) and the imaginal discs of the legs (Figs. 2b, 3c) are clearly visible.

After exposure to *Bti*, larvae became less active and behave erratically, with slower and slower movements, fewer trips to the surface to take oxygen and eventual death. During this process larvae become progressively softer and more flaccid. When compared to control larvae (Figs. 1c,f, 2a, 3a, 4a,b), according to the time of exposure to *Bti*, a progressive deformation occurs, and conspicuous empty spaces were observed between the cuticle and the internal structures. These changes are visible under a stereoscopic binocular microscope (Fig. 1d,e) and are clearly shown in rendered micro-CT images (Figs. 1h,j, 2b, 3b,c, 4c–f). Furthermore, when viewed in cross section, it is clear that in comparison to the untreated larvae, which have a flat ventral part (Fig. 4a,b), after exposure to *Bti*, the section becomes almost circular (Fig. 4e,f). In parallel, when compared to control larvae (Figs. 1c,f,g, 2a, 3a, 4a,b), the progressive cell lysis effect caused by *Bti* is observed as an increase in vacuoles and a progressive swelling of the midgut cell epithelia, in which an increase in thickness can be observed along with extensive openings in the walls of the midgut (Fig. 1i–k). These openings are observable in the 3D rendered images, in which the external surface of the midgut epithelium has been reconstructed as very conspicuous midgut transverse slits (Fig. 2b), as well as in the cross-sections of rendered images (Figs. 3c,d, 4c–g). In addition to these openings, vacuoles in the midgut epithelial cells are visible (Figs. 3d, 4f,g). Moreover, the dorsal tracheal trunks progressively collapse from a regular oblong section (Fig. 4a,b), reducing their lumina (Fig. 4e,f), until they become almost indistinguishable (Fig. 4g). After 6 h of exposure to *Bti*, the degradation was maximal, and larvae showed highly degraded midgut epithelial walls, an increase of cellular volume with vacuoles and openings and totally collapsed tracheal trunks (Figs. 1k, 3d, 4e–g).

## Discussion

The effect of *Bti* on larval movement observed here were previously reported in *Aedes albopictus*^26^ and *A. aegypti*^36^.

The micro-CT rendered 3D images shown in this work are comparable to the ones obtained with classical anatomical methods^33^. Thus, the 3D rendered images we obtained from *A. aegypti* allow the identification of anatomical structures and organs previously described for Culicidae mosquitoes in classic works such as those of Snodgrass^29^ or Christophers^30^ as well as more recent ones^19–21,26,31,37–39^. Even the histological images shown in those previous papers are comparable to the micro-CT rendered images shown in Figs. 3 and 4. In addition, the quality of rendered micro-CT images obtained is far superior to those obtained by Optical Coherence Tomography techniques, recently used to study mosquitoes^40^. We previously used Micro-CT to describe the anatomy of different insects^33,41–57^, but this is the first time, as far as we are aware, that this technology has been used to study the effects of a pathogen on its host.

A similar degradation of midgut epithelia observed in this work has been widely reported in other Diptera, particularly in Culicidae mosquitoes, after exposure to *Bti* toxins^18–20,22–26,28,37,39^. In fact, the first histological evidence of histopathological damage caused by *Bti* in *A. aegypti* was published by Charles & Barjac^19,20^, who determined and described how at the first signs, the fastest evolution occurs in the midgut. They observed that it was completely lysed 25 min after the addition of the toxin, whereas the cells in the subsequent areas were much less altered at this stage. The cells of the proventriculus did not appear to undergo any change, and the peritrophic membrane continued to be secreted without observable change. Lüthy & Wolfersberger^28^ reported that intracellular histopathological changes take place very fast, within a time frame of five to ten minutes. These are fully consistent with our observations as at 30 min after exposure to *Bti* we observed lysis of the midgut epithelial walls, clearly visible as conspicuous openings. Moreover, Clark^37^ described that after *Bti* exposure the epithelial cells present a degraded aspect with cytoplasmic extraction, an increase of cellular volume, secretory vesicles and vacuoles. In summary, damage consisted of holes and blisters in the membrane, with separation of cells, which is fully consistent with what we observed by micro-CT after progressive exposure to *Bti*.

Both the Cry and Cyt protein families produced by *Bt* have activity against insects of different orders by altering membranes. Specifically, Cyt toxins directly interact with membrane lipids^6^, affecting membrane permeability in insect cell lines, while Cry toxins kill cells by forming pores after receptor recognition and binding, leading to cell death by colloid osmotic lysis^28,58^. In our study, we observed the combined effect of both types of toxins, as *Bti* 4Q5 produces three Cry toxins (Cry4Aa, Cry4Ba and Cry11Aa) and one Cyt protein (Cyt1Aa), with possible production of Cyt2Ba, Cry1Ca and Cry10Aa^59^.

From a macroscopic point of view, after *Bti* exposure, alterations in the midguts result in osmotic shock and an accumulation of water in the larval body, creating empty space between the cuticle and internal structures, and the observed deformation of the abdomen that becomes roughly circular in section.

Regardless of the mechanisms that have been postulated in recent years as different models for lysis and cell death, currently the damage caused by the degradation of the epithelial walls of the digestive tract has been considered the main reason for the death of the larvae. Thus, traditionally, it has been considered that this destruction of the gut leads to the rapid cessation of feeding and subsequent death of the insect by inanition^59^. However, at least in *Ae. Agypti*’s case, gut malfunction and inanition could not produce death in such a short period of time. Moreover, the intestinal membrane is destroyed as the release of alkaline gastric juices into the hemolymph changes the pH and alkalinizes it. In insects, these changes have been shown to affect the functioning of the nervous system, even producing paralysis^60^. This is congruent with our observations. Thus, the collapse of cells and consequent damage of organs, alterations to the nervous system affecting movements and in extreme cases paralysis, certainly compromise the normal movements of the larvae towards the water surface and the proper positioning of the breathing siphon for gas exchange. In addition, the collapse of the tracheal trunks, first observed in this study, would certainly imply death due to a synergy of factors, including difficulty performing gas exchange correctly.

## Conclusions

The use of the micro-CT technique enabled us to make a complete reconstruction of the anatomy of fourth instar larvae of the yellow fever mosquito species *A. aegypti*, locating the actual position of internal structures and organs and comparing internal anatomical structures of healthy larvae with others after different times of exposure to *Bti* and comparing evidence of the damages. We also include detailed 3D micro-CT rendered images and Supplementary Video S1. This work represents the first complete micro-CT reconstruction of the internal anatomy of fourth instar larvae of *A. aegypti*, showing the damage caused by the action of *Bti*, such as the thickening of the epithelial cells of the midgut (enterocytes), the appearance of vesicles (vacuoles) and the separation of the cells generating the openings (holes and slits) that appear in the midgut minutes after exposure to *Bti*. These findings were consistent with the phenomena first described and illustrated by means of histological sections observed on slides. However, micro-CT has allowed us to obtain high quality rendered images of details of the larvae, some of which are equivalent to those previously obtained by optical microscopy, but we could see the entire animals instead of part of the larvae, with totally new perspectives of the 3D structures within the whole specimens.

Cell lysis alters different tissues and organs, with likely changes in the pH of the haemolymph by the release of gastric juices from the intestine. This would affect the normal functioning of the nervous system, producing the already known effect on the motility of the larvae. It would make it difficult for the larvae to reach the water surface and position the respiratory siphon in a correct way to break the surface tension of the water. These factors, together with the fact that micro-CT has shown that *Bti* produces an osmotic shock, creating extensive empty space and a collapse of the dorsal tracheal trunks, would certainly explain the death of the larvae, not by inanition as previously stated, but due to synergistic catastrophic multifactor processes. In addition, asphyxia would occur due to lack of adequate gas exchange. The approach used and the results obtained here open new perspectives for further research and prove that Micro-CT represents a strong methodology, not only to study insect anatomy but also to study the pathogenic effect of entomopathogens toward insects.

## Supplementary Information

Supplementary Legends.

Supplementary Video 1.

## Data Availability

The datasets generated and analyzed during the course of the study are available from J.A.-T. upon reasonable request.
