# Supplementary material for: Anatomical damage caused by Bacillus thuringiensis variety israelensis in yellow fever mosquito Aedes aegypti (L.) larvae revealed by micro-computed tomography
Source: Sci Rep. 2023 May 30;13:8759. doi: 10.1038/s41598-023-35411-1 (PMC10229604; doi:10.1038/s41598-023-35411-1)
Supplement: Supplementary file 1 — Supplementary Legends. [file 41598_2023_35411_MOESM1_ESM.docx]

**Supplementary information Video (mp4):**

**S1.** Animated 3D volume-rendered images showing anatomy of fourth instar *A. aegypti* larvae and evidencing damages caused by *B.* *thuringiensis* var. *israelensis* (*Bti*) by comparing a healthy larva with one after 1 h exposure to *Bti*.
